# Consumption of Ultra-Processed Foods and Biochemical Markers Predictive of Type 2 Diabetes Mellitus in a Self-Selected Pilot Sample of Muslim Adolescents in Melilla

**DOI:** 10.3390/foods15020319

**Published:** 2026-01-15

**Authors:** Miriam Mohatar-Barba, María López-Olivares, Emilio González-Jiménez, Aída García-González, Javier S. Perona, Carmen Enrique-Mirón

**Affiliations:** 1 Department of Nursing, Faculty of Health Sciences, Melilla Campus, University of Granada, 52005 Melilla, Spain; miriamb@ugr.es; 2 Instituto de Investigación Biosanitaria (ibs.GRANADA), 18014 Granada, Spain; 3 Department of Nutrition and Food Science, Faculty of Health Sciences, Melilla Campus, University of Granada, 52005 Melilla, Spain; mlopezolivares@ugr.es; 4 Department of Nursing, Faculty of Health Sciences, University of Granada, 18016 Granada, Spain; 5 Department of Food and Health, Institute of Fat-CSIC, Pablo de Olavide University Campus, Building 46, 41013 Seville, Spain; aida.garcia@csic.es (A.G.-G.); perona@ig.csic.es (J.S.P.); 6 HUM-613 Research Group, Department of Inorganic Chemistry, Faculty of Health Sciences, Melilla Campus, University of Granada, C/Santander s/n, 52005 Melilla, Spain; cenrique@ugr.es

**Keywords:** ultra-processed foods, adolescents, type 2 diabetes mellitus, religion, inflammation

## Abstract

The consumption of ultra-processed foods (UPFs) in adolescence is high due to their widespread availability and accessibility and has been linked to increased cardiometabolic risk. In the Autonomous City of Melilla, an environment with particular cultural and religious characteristics, it is relevant to analyze the relationship of UPFs with metabolic markers of type 2 diabetes mellitus. This is a cross-sectional pilot study on 31 Muslim adolescents aged 15 to 17 years. The NOVA food classification was used to identify UPFs. The final sample comprised Muslim adolescents because written consent for venous blood sampling was obtained only from Muslim families/legal guardians. Separate multiple linear regression models adjusted for sex were fitted to examine the associations between UPF intake (%E/day) and each cardiometabolic and inflammatory marker. Higher UPF intake was positively associated with BMI, body fat percentage, waist circumference, waist-to-height indicator (ICA), and fasting glucose after controlling for the false discovery rate (q < 0.05). Regarding the inflammatory component, Muslim girls had elevated levels of IL-7, IL-10, and IL-13, and Muslim boys had higher levels of MIP-1β. In addition, IL-8 correlated positively with waist circumference, BMI, and the HDL/LDL ratio, while MCP-1 was negatively associated with Apo A1, total cholesterol, and HDL. In this exploratory pilot study, higher intake of UPF appears to be associated with greater central adiposity and higher fasting glucose; these hypothesis-generating findings warrant confirmation in larger, representative samples and may inform culturally adapted nutritional screening in Melilla.

## 1. Introduction

Adolescence is a stage in which skipping meals, irregular eating patterns, and a preference for high-calorie, nutrient-poor foods are common. These behaviors represent a departure from the Mediterranean dietary pattern and are largely driven by globalization and the progressive adoption of a Western dietary model [1]. Although health-promotion campaigns exist, the ubiquity and ready access to these products in high-income countries have eroded young people’s diet quality and health [1,2].

The consumption of ultra-processed foods (UPFs) is increasing steadily worldwide. Although it is higher in high-income countries, it is also growing in low-income countries, where price determines product choice [3]. In Spain, 32.2% of the total energy consumed by children and young people comes from the intake of UPFs [4]. Furthermore, a study of 560 Spanish adolescents aged 14 to 17 in the region of Catalonia showed high consumption of UPFs, with more than 50% of participants consuming sugary cookies, chocolate snacks, processed meats, sugary drinks, flavored yogurts, and sauces on a daily basis [5].

Similarly, in a study of 590 adolescents aged 15 to 17 in Melilla, it was observed that the daily energy intake (kcal) from consumption of ultra-processed products was 40.5% among Christian schoolchildren and 59.1% among Muslims. In addition, in that cohort, Muslim schoolchildren had a higher mean total energy intake (2640.2 kcal/day) compared to Christians (2417.7 kcal/day) [6]. These values refer to the larger cohort and are provided for context; total energy intake in the present pilot subsample is reported separately in the Results section.

From a nutritional point of view, dietary exposure to UPFs is characterized by their low fiber, protein, and micronutrient content, and by being very energy-dense and rich in saturated and trans fats, added sugars, and sodium [7,8,9]. In addition, they often include flavorings, colorings, emulsifiers, and other additives that are particularly appealing to adolescents. These characteristics promote the development of noncommunicable diseases such as type 2 diabetes mellitus (T2DM), metabolic syndrome, and cardiovascular disease, among others [2].

Several factors are considered to induce T2DM, including obesity and the resulting metabolic dysfunction of abdominal adipose tissue, which leads to an alteration in triglyceride (TG) storage, an increase in the rate of lipolysis, and the secretion of proinflammatory cytokines, like alpha tumor necrosis factor (TNF-α), interleukin 6 (IL-6), and interleukin 1β (IL-1β) by visceral adipose tissue [10].

Obesity is a chronic disease characterized by excess body fat and adverse health effects. In this context, obesity in childhood and adolescence has become the most prevalent form of malnutrition globally. The 2025 Child Nutrition Report indicates that it already affects 188 million children and adolescents (approximately 1 in 10) and exceeds underweight statistics in most regions. In addition, it estimates 391 million individuals are overweight (about 1 in 5) and warns of the role of UPFs and their marketing to minors [11]. These data underscore the urgency of studying UPF exposure in adolescents and its cardiometabolic impact on our environment.

Despite the growing literature linking UPF intake to adiposity and metabolic risk, data on biochemical markers related to early T2DM risk (e.g., fasting glucose, lipoproteins, and inflammatory cytokines) in adolescents from Melilla are scarce. Moreover, the previously published cohort study in Melilla provided dietary and anthropometric information, but venous blood biomarkers were not available for most participants. Therefore, we conducted a feasibility-driven cross-sectional pilot nested within this cohort to explore associations between UPF intake (%E/day) and markers of adiposity, glycemia, lipid profile, and inflammatory cytokines in adolescents who provided written consent for venous blood sampling (which in our setting resulted in an exclusively Muslim subsample). We hypothesized that higher UPF intake would be associated with risk factors for developing T2D.

## 2. Materials and Methods

### 2.1. Study Design and Subjects

A cross-sectional design was applied to a purposive sample of 31 Muslim adolescents, 17 boys and 14 girls, enrolled in compulsory secondary education in the Autonomous City of Melilla; the average age was 15.67 ± 0.62 years. This exploratory pilot study used a purposive (non-probability) sampling approach; therefore, the sample is not intended to be representative of the overall adolescent population in Melilla.

### 2.2. Data Collection

**Figure 1:**
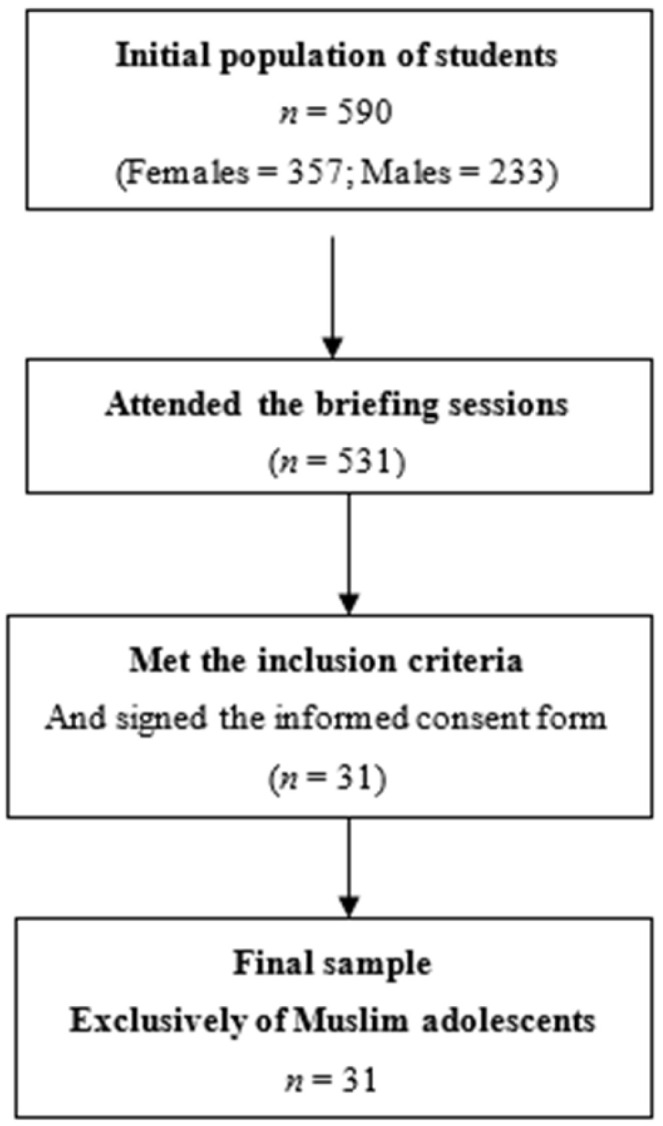
Flow diagram of the recruitment process.

The selection of participants in this pilot study was based on an initial cohort of 590 adolescents included in a general study conducted in Melilla [7]. From this initial cohort, and considering the adolescents’ nutritional status we purposively invited adolescents aiming to include participants across nutritional status categories (underweight, normal weight, overweight and obesity), according to the cut-off points proposed by Cole et al. [12], which define overweight as above the 85th percentile and obesity as above the 95th percentile according to age and sex. However, because inclusion ultimately depended on written parental/legal-guardian consent for venous blood sampling, the final sample should be considered a convenience, self-selected pilot sample. As a result, the intended distribution across nutritional status categories within sex was not fully achieved (e.g., no obese girls participated). Subsequently, meetings were held in each of the educational centers with the parents and/or guardians responsible in order to explain the characteristics of the pilot study and obtain written consent. Venous blood sampling was offered to the invited adolescent; consent for blood collection was obtained only from Muslim families/legal guardians, resulting in a final pilot sample composed exclusively of Muslim adolescents (*n* = 31). Furthermore, since participation required parental/legal-guardian consent for venous blood sampling, the possibility of self-selection cannot be ruled out. The reasons for non-consent to venous blood sampling were not systematically collected. Therefore, we cannot determine the specific factors underlying differential consent across groups in this setting. Figure 1 summarizes the recruitment process, including the number of adolescents who did not meet these criteria or did not provide consent for blood sampling.

In May 2024, informational sessions were organized at each center for parents or legal representatives, who were briefed on the tests and questionnaires required for their children’s participation. The following month, every participant completed anthropometric and body-composition assessments and a diet evaluation; blood was then drawn and forwarded to the Instituto de la Grasa (Seville, CSIC) for laboratory analysis.

The study was conducted in accordance with the principles of the Declaration of Helsinki, and ethical approval was obtained from the corresponding institutional review board. Further details are provided in the Institutional Review Board Statement section [13].

### 2.3. Blood Pressure

Blood pressure (BP) was measured using a previously calibrated sphygmomanometer and a Littmann^®^ stethoscope, (3M Company, St. Paul, MN, USA) following the recommendations of the Subcommittee on Professional and Public Education of the Council for High Blood Pressure Research of the American Heart Association [14]. To take the measurement, the participants remained seated for five minutes, with their back supported, feet flat on the floor, and wrist relaxed at heart level, in silence and relaxed.

### 2.4. Dietary Intake

Each participant completed a 44-item food frequency questionnaire (FFQ) to record how often they consumed each food (daily, weekly, or monthly) [15]. In addition, a 72 h dietary record (Thursday, Friday, and Saturday) was used, collecting food consumed over three days, to identify possible variations in consumption frequency between weekdays and weekends [16]. Both instruments have been previously validated for use in adolescent populations.

The record was completed by trained researchers through face-to-face interviews, requesting information on foods, beverages, and supplements consumed during those three days. Standardized household measures and reference images were used to estimate portions.

Energy intake and macro- and micronutrient calculations were estimated using the calculator provided by the Valladolid Institute of Endocrinology and Nutrition (IENVA) (https://calcdieta.ienva.org/tu_menu.php, accessed on 1 February 2024). The main exposure variable (UPF intake, %E/day) was derived from the 72 h dietary record. Specifically, foods and beverages recorded over the three days were classified according to NOVA; total daily energy intake from NOVA group 4 items was divided by total daily energy intake and averaged across the three recorded days to obtain %E/day. The FFQ was used to characterize usual frequency of consumption and to support the identification of commonly consumed UPF groups, but it was not used in the quantitative calculation of %E/day.

Foods and beverages from the FFQ and the 72 h record were also classified according to the NOVA classification, which groups foods by degree and purpose of processing: group 1, unprocessed or minimally processed foods (fruits, vegetables, milk, meat); group 2, processed culinary ingredients (salt, sugar, oils, butter); group 3, products processed by adding ingredients from group 2 to those from group 1 (bread, fresh cheese, canned vegetables); and group 4, ultra-processed foods (mixtures of multiple industrial ingredients and additives—e.g., high fructose corn syrup, colorants, emulsifiers, preservatives)—such as sugary soft drinks, frozen meals, or packaged bread. In the 72 h record, each food was classified using NOVA, and the IENVA calculator was used to estimate the calories and key nutrients from ultra-processed foods consumed by each participant.

### 2.5. Anthropometric Measurements

Anthropometric measurements (height, weight, waist, and hip circumference) were taken following the standards of the International Society for the Advancement of Kinanthropometry (ISAK) [17]. Body composition was assessed using TANITA^®^ SC-330 (Tanita Corporation, Tokyo, Japan) bioimpedance analysis.

### 2.6. Physical Activity

Physical activity was assessed using the short form of the International Physical Activity Questionnaire (IPAQ) for individuals aged 15–69 years [18]. Total physical activity was estimated in MET-minutes per week following the IPAQ scoring protocol, and participants were classified into low, moderate, or high physical activity levels according to standard cut-off points.

### 2.7. Biochemical Analysis

Venous blood samples were taken from participants in the pilot study (31 subjects) by qualified professionals after an overnight fast (12 h). At 8:00 a.m., 10 mL of blood was drawn by venipuncture in the antecubital fossa of the right arm using a disposable vacuum blood collection tube. Subsequently, and taking into account the appropriate preservation measures, the blood samples collected were immediately processed and then sent to the Instituto de la Grasa, a center belonging to the Spanish National Research Council (CSIC), in Seville, where the study was carried out to analyze the fatty acid composition of blood serum, lipoproteins directly involved in the development of atherosclerosis, such as very low-density lipoproteins (VLDL) and erythrocyte membrane, as well as the plasma concentrations of different pro- and anti-inflammatory interleukins. Likewise, circulating lipids were determined at the Laboratory of Analysis and Clinical Biochemistry of the Virgen del Rocío University Hospital in Seville.

Serum and plasma were aliquoted and stored at −80 °C until analysis. Routine circulating lipids (total cholesterol, triglycerides, and HDL-C) and fasting glucose were determined at the Laboratory of Analysis and Clinical Biochemistry of Virgen del Rocío University Hospital using standard enzymatic methods. LDL-C was calculated using the Friedewald equation when applicable, and VLDL-C was estimated as triglycerides/5 (mg/dL). Apolipoproteins (Apo A1 and Apo B) and lipoprotein(a) were measured using routine immunoassays according to the hospital laboratory standard operating procedures.

Fatty acid composition in serum and erythrocyte membranes was analyzed at the Instituto de la Grasa (CSIC, Seville). Total lipids were extracted, fatty acid methyl esters were prepared, and fatty acids were quantified by gas chromatography (GC-FID) using external standards for identification. Results are expressed as the relative percentage of total identified fatty acids.

Plasma cytokines (including IL-7, IL-8, IL-10, IL-13, MCP-1, and MIP-1β) were quantified at the Instituto de la Grasa using a commercially available immunoassay (MILLIPLEX® MAP Human Cytokine/Chemokine Panel; Merck KGaA, Darmstadt, Germany). Samples were analyzed in duplicate, and assay sensitivity (lower limits of detection) and intra/inter-assay coefficients of variation are available from the authors upon request.

### 2.8. Religion

Each participant indicated their religious practice (Islam or Christianity). To measure this variable, the Religious Attitudes Questionnaire developed and validated by Orozco-Parra et al. [19] was used.

### 2.9. Statistical Analysis

For physical and sociodemographic characteristics, continuous variables were expressed as mean ± standard deviation, and qualitative variables as frequencies and percentages. Since quantitative variables did not follow a normal distribution (Shapiro–Wilk test), the Mann–Whitney U test was used.

Categorical variables were analyzed with Fisher’s exact test. The number of participants who consumed UPFs at different times was evaluated using contingency tables and reported as frequency (percentage).

WHO recommended intakes were used to estimate UPF intake (low intake or high intake) [20]. Statistical analyses were performed using SPSS version 28.0 [21] using two-sided tests. To address multiplicity, we pre-specified a primary family of outcomes (BMI, body fat percentage, waist circumference, waist-to-height indicator [ICA], and fasting glucose) and controlled the false discovery rate using the Benjamini–Hochberg procedure (q < 0.05). All other outcomes were considered exploratory and are presented with unadjusted *p*-values.

Associations between UPF intake (%E/day, continuous) and each cardiometabolic and inflammatory outcome were examined using separate multiple linear regression models. For each outcome, UPF intake was entered as the main independent variable and sex was included as the only covariate (Enter method) to keep the models parsimonious, given the small sample size. No additional covariates were included to reduce the risk of model overfitting; therefore, these analyses should be interpreted as exploratory (hypothesis-generating).

As this was an exploratory pilot study, no a priori sample size calculation was performed, and the sample size was determined by feasibility (availability and consent for venous blood sampling). Missing data was handled using complete-case analysis. No imputation was performed. The pilot effect estimates and variability will be used to inform the sample size calculation for a future adequately powered study.

On the other hand, given the small pilot sample size and the non-normal distribution of several variables, we additionally examined non-parametric associations using Spearman’s rank correlations as a sensitivity analysis. Cytokine analyses were treated as exploratory and are presented using descriptive statistics, sex comparisons, and Spearman correlations rather than relying on regression-based inference. Overall, regression results are reported with caution as hypothesis-generating estimates rather than confirmatory findings.

## 3. Results and Discussion

A pilot study was conducted to explore associations between UPF consumption and metabolic markers of T2DM risk among adolescents. The sample consisted of 31 participants (5.1% of the total sample), whose mean age was 15.67 ± 0.62 years, with 45.16% being girls and 54.83% boys. The entire sample stated that they were Muslim.

Analyses were conducted on participants with complete data for the variables included in each model (complete-case analysis). No missing data were observed for the variables included in the regression models.

### 3.1. Sociodemographic, Physical, and Biochemical Characteristics of the Participating Students

Table 1 summarizes the anthropometric characteristics, nutritional status and blood pressure of the pilot sample by sex. Overall, boys had higher height and weight, whereas girls showed a higher percentage of body fat, as expected during mid-adolescence. Overweight and obesity were present in both sexes, indicating an unfavorable nutritional profile in this small sample of Muslim adolescents living in Melilla.

Table 2 presents the nutritional characteristics (UPF intake and total energy intake), physical activity and biochemical variables stratified by sex. In descriptive analyses, mean lipid concentrations differed between boys and girls (Table 2). However, given the small, self-selected pilot sample, these sex differences should be interpreted cautiously and considered hypothesis-generating rather than confirmatory. Girls tended to show a more adverse lipid profile than boys, with higher mean triglycerides, very low-density lipoprotein cholesterol (VLDL-c), high-density lipoprotein cholesterol (HDL-c) and total cholesterol/HDL-c ratio. These differences suggest that, despite their higher body fat being partly explained by pubertal development, some girls may already exhibit early atherogenic alterations. In the context of adolescence, such patterns are worrisome because they may track into adulthood and contribute to an increased cardiometabolic risk. In the present pilot sample, mean total energy intake was 2382.11 ± 449.11 kcal/day overall, 2539.63 ± 534.52 kcal/day in boys, and 2190.82 ± 204.88 kcal/day in girls (Table 2).

First, it is important to emphasize that this is a feasibility-driven, cross-sectional pilot study with a very small, self-selected convenience sample (*n* = 31) nested within a larger cohort. Consequently, statistical power is limited, estimates are unstable, and findings should be interpreted strictly as exploratory and hypothesis-generating rather than confirmatory. Throughout the Discussion, we therefore focus on patterns of association and effect direction, and we avoid causal or generalisable statements.

Taken together, the descriptive results highlight that, even in this small pilot sample, an important proportion of Muslim adolescents presented excess adiposity and an unfavorable biochemical profile, which provides a relevant context for examining the potential impact of UPF intake.

To our knowledge, our study is the first to analyze the relationship between UPF consumption and risk factors for T2DM and inflammation in Muslim adolescents.

In descriptive analyses, mean lipid concentrations differed between boys and girls (Table 2). However, given the very small, self-selected pilot sample and the lack of power for sex-stratified inference, these differences should be interpreted cautiously and considered hypothesis-generating only. Such patterns may reflect pubertal development, hormonal changes, and other unmeasured factors rather than robust sex-specific effects. Larger, representative studies are needed before drawing conclusions about sexual dimorphism in lipid metabolism in this setting.

Girls in our study had significantly higher VLDL and triglyceride concentrations than boys. These findings are clinically relevant because elevated VLDL promotes lipid accumulation in the vascular endothelium and may favor early atherogenesis. Sexual dimorphism in lipid metabolism during adolescence, probably associated with pubertal hormonal changes, may partly explain these sex differences [22].

The presence of significantly elevated VLDL levels in girls is clinically relevant, as high concentrations of these lipoproteins can promote the accumulation of lipids in the vascular endothelium, favoring the early development of atherogenesis. Furthermore, according to Linton et al. [23], during adolescence, VLDL particles have structural characteristics that make them particularly atherogenic, with a greater capacity for infiltration into the arterial intima.

As mentioned above, in our study, girls had significantly higher levels of HDL-C than boys. These results are similar to those described by Eissa et al. [24], who, in their study of 633 American adolescents, showed that in boys aged 8 to 18 years, serum HDL-C levels decreased by 15%, while girls maintained their levels. Similarly, Cho & Kim [25], in their study of Korean adolescents, observed that boys between the ages of 10 and 19 had significantly lower HDL cholesterol levels than girls.

These changes in lipid metabolism in boys suggest that puberty is accompanied by a redistribution of HDL-C and a transiently more atherogenic profile. This pattern could contribute to a higher cardiometabolic risk in males during adolescence and later in adulthood [22,24,25].

### 3.2. Association Between UPF Intake and Risk Factors for Developing T2DM and Inflammation

Table 3 summarizes the results of separate sex-adjusted multiple linear regression models, one per outcome. After controlling the false discovery rate (Benjamini–Hochberg) across the pre-specified primary outcomes (BMI, body fat percentage, waist circumference, ICA, and fasting glucose), higher UPF intake remained associated with higher adiposity markers and fasting glucose (q < 0.05). All other outcomes in Table 3 are considered exploratory and are therefore presented with unadjusted *p*-values.

In this self-selected pilot sample, higher UPF intake was observed to be positively associated with adiposity indicators (BMI, body fat percentage, waist circumference, and ICA) and fasting glucose after FDR control across the pre-specified primary outcomes (q < 0.05). These exploratory associations are consistent in direction with prior literature, but given the pilot design and selection bias, they should be interpreted as hypothesis-generating rather than confirmatory [26,27,28,29]. Similarly, Juul et al. [30] observed in young American adults that UPF consumption was associated with higher BMI and waist circumference values.

On the other hand, UPF consumption showed a positive association with fasting glucose. Regarding blood pressure, UPF intake was inversely associated with systolic blood pressure (SBP) in the exploratory analyses (unadjusted *p* = 0.018); however, SBP was not part of the pre-specified primary outcome family, and the direction of this association is counterintuitive. Therefore, this finding should be interpreted with caution and confirmed in larger, representative samples. Overall, in this pilot study, the most consistent associations after multiplicity control were observed for adiposity indicators and fasting glucose. These results partially coincide with studies such as that conducted by Mendonça et al. [31], in which UPF intake was associated with increased insulin resistance, although such changes could be mediated by the action of sex hormones during adolescence. These findings contrast with our results, probably due to the predominance of a visceral fat accumulation pattern or the possible influence of estrogens on tissue sensitivity to insulin [27].

Our findings in Muslim adolescents from Melilla are consistent with global trends linking ultra-processed food consumption to adiposity and metabolic risk [32]. This suggests that cultural differences in dietary patterns and the traditional Mediterranean context may not fully protect against the metabolic consequences of ultra-processed products. In this sense, our data extends previous evidence by documenting these associations in a scarcely studied population group living in a multicultural border city.

Finally, the association between pro- and anti-inflammatory cytokines and risk factors for T2DM was examined. The correlation coefficients between cytokine concentrations and anthropometric and metabolic variables are shown in Table 4. In general, higher levels of pro-inflammatory cytokines were found to be positively correlated with markers of adiposity and insulin resistance, supporting the concept that low-grade chronic inflammation is involved in the early development of metabolic alterations in adolescents.

The study of proinflammatory and anti-inflammatory cytokine levels has gained interest as a potential marker of T2DM in different populations worldwide [10]. Scientific evidence suggests that low-grade chronic inflammation, together with increased baseline levels of inflammatory mediators, plays a key role in the pathogenesis of T2DM [32].

We observed sex differences in cytokine concentrations in this pilot sample; however, cytokine analyses were exploratory, and the study was not powered to evaluate effect modification by sex. Therefore, these sex-related patterns should be interpreted cautiously and confirmed in larger studies before any inference about sexual dimorphism is made. On the one hand, serum levels of IL-7, IL-10, and IL-13 were higher in girls, which could reflect greater anti-inflammatory activity or a different modulation of the immune system compared to boys. These findings are consistent with previous studies that point to sex differences in circulating cytokine levels, possibly influenced by sex hormones such as estrogens, which have the ability to modulate the immune response [33]. On the other hand, our results show higher levels of MIP-1β among boys, a proinflammatory cytokine synthesized by macrophages and lymphocytes, involved in the immune response and associated with obesity and insulin resistance. This finding is consistent with the studies by Ognjanovic et al. [34] and Roth et al. [35], who also describe higher levels of MIP-1β in adolescent boys with greater visceral adiposity and insulin resistance.

In exploratory analyses, IL-7 showed an inverse correlation with SBP. The biological meaning of this observation is uncertain, and we did not measure renin–angiotensin system markers; therefore, no mechanistic conclusions can be drawn. This finding should be treated as hypothesis-generating and confirmed in larger, adequately powered studies. However, this association could indicate that elevated IL-7 levels in adolescents contribute to a more favorable cardiovascular profile [36,37].

Regarding IL-8, there is a positive correlation with waist circumference, BMI, and HDL/LDL ratio. This association with abdominal obesity and lipid abnormalities is consistent with the results of other studies conducted with adolescent populations with insulin resistance [38]. An elevated cHDL/LDLc ratio suggests an atherogenic profile, reinforcing the role of IL-8 as a bridge between inflammation and cardiovascular risk in adults, although scientific evidence in adolescents is currently limited [39]. These results support the use of IL-8 as an early biomarker of metabolic risk in adolescents with excess adiposity.

Our results show significant correlations between elevated levels of MCP-1 and an atherogenic lipid profile in adolescents, characterized by a decrease in APO A1, total cholesterol, and HDL-C. These findings are consistent with previous work showing that, in obese individuals, adipose tissue macrophage recruitment and increased secretion of MCP-1 and other pro-inflammatory cytokines contribute to a state of low-grade chronic inflammation and insulin resistance, thereby increasing the risk of T2DM [40,41,42,43].

### 3.3. Strengths and Limitations

This study has strengths and limitations. A key strength of this work is its feasibility contribution: it demonstrates the practical implementation of dietary assessment together with fasting blood biomarker collection and laboratory profiling in adolescents in Melilla, a setting that is rarely represented in biomarker-based nutritional studies and that is generally excluded from epidemiological studies conducted at the national level. Therefore, our results provide relevant and novel information about the Muslim adolescent population in Melilla. However, because the resulting sample is small and self-selected, the present findings are intended primarily to generate hypotheses and to inform the design of future, adequately powered studies.

On the other hand, this study has several limitations. First, it is an exploratory pilot study with a small sample size, which limits statistical power and the generalizability of the findings. Although regression models were intentionally restricted to UPF intake and sex to reduce overfitting, residual confounding cannot be ruled out.

The paramount limitation is the very small, non-probability, self-selected sample (*n* = 31), which substantially limits statistical power and precludes drawing reliable, generalisable conclusions about associations. In addition, participation required written consent for venous blood sampling, which may have introduced self-selection bias. In our setting, only Muslim families provided consent for blood collection, which limits external validity and precludes extrapolation of these findings to adolescents from other religious/cultural backgrounds. Moreover, because reasons for refusal were not systematically documented, we could not formally assess whether differential consent was driven by specific logistical, informational, or cultural factors. This is particularly relevant for the analyses involving inflammatory cytokines.

Therefore, the sample size was not calculated a priori, as this was a feasibility-based pilot study. Furthermore, the requirement for written consent for venous blood sampling and the use of complete case analyses may have contributed to selection bias and limited the representativeness of the findings.

Second, dietary intake was self-reported using an FFQ and a 72 h dietary record, which are subject to recall and reporting bias. These limitations may have attenuated or biased some of the observed associations, and our findings should therefore be interpreted as hypothesis-generating. In addition, because multiple outcomes were examined, we controlled the false discovery rate for a pre-specified primary family of outcomes; however, findings from exploratory comparisons should be interpreted cautiously. Overall, this study should be interpreted as exploratory and hypothesis-generating; its main value is to highlight feasibility considerations and candidate outcomes for future, larger, representative studies in Melilla.

## 4. Conclusions

In this self-selected, feasibility-driven pilot sample of Muslim adolescents in Melilla, higher intake of UPF appears to be associated with higher indicators of adiposity and fasting glucose. However, these observations are exploratory and non-generalizable, and they do not support causal inference.

The findings justify the need for larger, adequately powered studies using representative sampling across religious/cultural groups in Melilla, with systematic documentation of reasons for participation/non-participation in blood sampling. Future work should also clarify the role of inflammatory cytokines using pre-specified hypotheses and appropriate multiplicity control.

## Figures and Tables

**Table 1:** Anthropometric characteristics, nutritional status, and blood pressure of the pilot sample by sex.

		All (*n* = 31)	Boys (*n* = 17)	Girls (*n* = 14)
Age (years)		15.67 ± 0.62	15.76 ± 0.66	15.51 ± 0.64
Height (cm)		167.87 ± 8.70	171.05 ± 9.20	164.00 ± 6.40
Weight (kg)		63.09 ± 15.63	68.13 ± 18.63 **	56.97 ± 7.95
BMI (kg/m^2^)		22.19 ± 4.69	23.16 ± 5.95 **	21.00 ± 2.11
**Nutritional status**				
	Underweight	6	6	0
	Normal weight	17	4	13 **
	Overweight	5	4	1
	Obese	3	3	0
Waist circumference (cm)		72.53 ± 9.96	76.05 ± 11.04 *	68.25 ± 6.53
Hip circumference (cm)		97.58 ± 10.35	98.88 ± 13.11 **	96.00 ± 5.56
WHI *		0.74 ± 0.06	0.77 ± 0.06	0.71 ± 0.05
WHT **		0.43 ± 0.05	0.44 ± 0.05 *	0.41 ± 0.03
**Cardiometabolic risk**				
	No risk (ICA < 0.5)	29	15	14
	At risk (ICA > 0.5)	2	2	--
Fat mass (kg)		12.93 ± 8.06	13.32 ± 10.53 **	12.47 ± 3.58
Body fat (%)		19.18 ± 9.71	17.00 ± 11.69	21.82 ± 5.96 *
Lean mass (kg)		50.53 ± 11.39	55.42 ± 11.82 *	44.60 ± 7.65
Muscle mass (kg)		47.60 ± 10.47	52.05 ± 10.87 *	42.20 ± 7.08
Heart rate		78.48 ± 9.85	79.29 ± 11.53	77.50 ± 7.66
SBP (mmHg)		115 ± 17.63	120.35 ± 16.78	109.85 ± 17.49
DBP (mmHg)		74.06 ± 13.90	76.00 ± 14.68	71.71 ± 13.04
MAP (mmHg)		94.83 ± 14.32	98.17 ± 13.61	90.78 ± 14.60
**BP level**				
	Normal	26	13	13
	Prehypertensive	3	2	1
	Hypertensive	2	2	--

Note. Data are presented as mean ± SD for continuous variables and as absolute counts *(n*) for categorical variables. Given the small, non-probability pilot sample, sex-stratified comparisons are descriptive and should be interpreted cautiously. BMI: Body mass index; WHI: Waist–hip index; WHT: Waist–height index; SBP: Systolic blood pressure; DBP: Diastolic blood pressure; MAP: Mean arterial pressure. * *p* < 0.05; ** *p* < 0.001.

**Table 2:** Dietary intake, physical activity, and biochemical characteristics of the pilot sample by sex.

Variables		All (*n* = 31)	Boys (*n* = 17)	Girls (*n* = 14)
Ultra-processed food intake (% total energy)		50.80 ± 11.60	51.47 ± 8.37	50.00 ± 14.94
Total energy intake (kcal)		2382.11 ± 449.11	2539.63 ± 534.52 *	2190.82 ± 204.88
Physical activity (min/day)		67.25 ± 45.15	93.92 ± 71.21 **	34.88 ± 19.10
**Lipid profile**				
	TG (mg/dL)	67.90 ± 30.58	60.05 ± 18.57	77.42 ± 39.43 *
	TC (mg/dL)	141.48 ± 24.80	138.94 ± 24.03	144.57 ± 26.27
	VLDLc (mg/dL)	13.58 ± 6.11	12.01 ± 3.71	15.48 ± 7.88 *
	LDLc (mg/dL)	86.61 ± 20.77	85.17 ± 19.80	88.35 ± 22.53
	HDLc (mg/dL)	47.34 ± 9.89	46.64 ± 7.45	49.64 ± 12.54 *
	Non-HDL cholesterol (mg/dL)	94.83 ± 20.51	92.29 ± 19.48	97.92 ± 22.03
	CT/HDLc ratio	3.12 ± 0.68	2.99 ± 0.35	3.27 ± 0.94 *
	HDLc/LDLc ratio	0.56 ± 0.16	0.56 ± 0.10	0.56 ± 0.21
Glucose (mg/dL)		80.13 ± 15.03	73.65 ± 7.50	88.00 ± 18.19
Insulin (µIU/mL)		8.43 ± 4.65	6.83 ± 4.11	10.24 ± 4.68 *
C-peptide (ng/mL)		1.75 ± 1.74	1.25 ± 0.57	2.37 ± 2.42
Lipoprotein (a) (mg/dL)		14.01 ± 18.20	13.42 ± 21.96	14.73 ± 13.05
Apo A1 (mg/dL)		86.13 ± 29.57	82.99 ± 23.22	89.95 ± 36.41
Apo B (mg/dL)		37.66 ± 11.63	36.07 ± 11.40	39.60 ± 12.03
Apo B/Apo A1		0.45 ± 0.13	0.43 ± 0.07	0.48 ± 0.17 *

Note. Data are presented as mean ± SD. Given the small, non-probability pilot sample, sex-stratified comparisons are descriptive and should be interpreted cautiously. TG: Triglycerides; TC: Total cholesterol; VLDL-C: Very low-density lipoprotein cholesterol; LDL-C: Low-density lipoprotein cholesterol; HDL-C: High-density lipoprotein cholesterol; Apo A1: Apolipoprotein A1; Apo B: Apolipoprotein B. * *p* < 0.05; ** *p* < 0.001.

**Table 3:** Associations between determinants of risk factors for the development of DM2 and the intake of UPFs using multiple linear regression analysis.

	β	IC95%	*p*	Q-Value (FDR)
IMC (kg/m^2^)	0.105	0.070, 0.141	0.025	0.041
Grasa corporal (%)	0.061	0.002, 0.120	0.041	0.041
Perímetro cintura (cm)	0.017	0.002, 0.031	0.024	0.041
ICA	0.015	0.002, 0.028	0.023	0.041
TAS (mmHg)	−0.210	−0.530, −0.120	0.018	-
TAD (mmHg)	0.100	−0.080, −0.280	0.274	-
TG (mg/dL)	1.050	−0.013, 0.072	0.096	-
CT (mg(dL)	−0.330	−0.124, 0.321	0.364	-
c-HDL (mg/dL)	−0.351	−0.239, −0.125	0.134	-
c-LDL (mg/dL)	−0.117	−0.830, 0.429	0.552	-
Apo A1 (mg/dL)	−0.005	−0.056, −0.014	0.334	-
Apo B (mg/dL)	0.001	−0.020, −0.019	0.476	-
Glucosa (mg/dL)	0.032	0.022, 0.620	0.034	0.041
Insulina (µUI/mL)	0.028	−0.021, 0.125	0.345	-

Note. BMI: Body mass index; WHR: Waist-to-height ratio; SBP: Systolic blood pressure; DBP: Diastolic blood pressure; MAP: Mean arterial pressure; TG: Triglycerides; TC: Total cholesterol; LDL-C: Low-density lipoprotein cholesterol; HDL-C: High-density lipoprotein cholesterol; Apo A1: Apolipoprotein A1; Apo B: Apolipoprotein B. Each row corresponds to a separate regression model adjusted for sex. Q-values are reported only for the pre-specified primary outcomes after Benjamini–Hochberg FDR correction; all other outcomes are exploratory and shown with unadjusted *p*-values.

**Table 4:** Association between pro- and anti-inflammatory cytokine levels and risk factors for T2DM.

	IL-1β	IL-7	IL-8	IL-10	IL-12p	IL-17	MCP-1	MIB-1B
SBP (mmHg)	−0.140	−0.541 *	0.257	−0.191	−0.158	−0.007	0.018	--
DBP (mmHg)	−0.059	−0.185	0.099	−0.010	−0.097	−0.106	−0.145	0.014
Waist circumference (cm)	0.156	−0.258	0.440 *	−0.264	−0.001	−0.069	0.199	0.099
WHT	−0.077	−0.194	0.471 *	−0.175	0.109	−0.073	0.136	0.148
BMI (kg/m^2^)	−0.081	−0.183	0.468 *	−0.167	0.010	−0.158	0.088	0.188
Physical activity (min/day)	−0.174	−0.208	−0.005	−0.155	−0.200	0.139	−0.008	0.063
Ultra-processed food intake (% total energy)	−0.101	0.224	0.038	0.026	0.105	−0.056	0.142	0.180
Total energy intake (kcal)	0.035	−0.048	0.053	−0.090	0.024	−0.027	0.083	0.078
ApoA1	−0.003	0.104	0.004	0.246	0.274	−0.166	−0.587 *	0.113
Apo B	−0.092	−0.066	−0.239	0.054	−0.027	−0.224	−0.221	0.061
Apo B/Apo A1	−0.099	−0.229	−0.272	−0.266	−0.461 *	−0.095	0.325	−0.066
TC	0.042	0.219	−0.314	0.170	−0.016	−0.095	−0.516 *	−0.190
HDLc	0.027	0.287	0.078	0.241	0.202	0.028	−0.689 **	−0.178
LDLc	0.004	0.125	−0.536 *	0.034	−0.197	−0.126	−0.270	−0.146
HDLc/LDLc ratio	0.028	0.051	0.645 **	0.080	0.285	0.087	−0.242	0.022
Glucose (mg/dL)	0.084	0.065	−0.181	0.132	−0.075	−0.129	−0.058	−0.147
Insulin (µIU/mL)	0.134	0.113	−0.193	0.189	−0.021	−0.125	−0.059	0.051

Note. Statistics used: Spearman’s coefficient (rs). ** *p* < 0.001 (two-tailed). * *p* < 0.05 (two-tailed). Given the number of correlations tested, results should be interpreted as exploratory; *p*-values are unadjusted for multiple comparisons.

## Data Availability

The original contributions presented in this study are included in the article. Further inquiries can be directed to the corresponding author.
